# Correction: Automatic hoof-on and -off detection in horses using hoof-mounted inertial measurement unit sensors

**DOI:** 10.1371/journal.pone.0236138

**Published:** 2020-07-09

**Authors:** M. Tijssen, E. Hernlund, M. Rhodin, S. Bosch, J. P. Voskamp, M. Nielen, F. M. Serra Braganςa

There are errors in the Funding statement. The correct Funding statement is as follows: Swedish-Norwegian Foundation for Equine Research (https://hastforskning.se/) provided funding for this study in the form of a grant awarded to author MR (H-17-47-303). Indirect support was provided through salaries by the home institutions of all co-authors. Inertia-Technology B.V. provided support in the form of salary for author SB. Rosmark Consultancy provided support in the form of salary for author JPV. The specific roles of these authors are articulated in the ‘author contribution’ section. The funders did not have any additional role in the study design, data collection and analysis, decision to publish, or preparation of the manuscript.
