# Supplementary material for: Automatic hoof-on and -off detection in horses using hoof-mounted inertial measurement unit sensors
Source: PLoS One. 2020 Jun 3;15(6):e0233266. doi: 10.1371/journal.pone.0233266 (PMC7269263; doi:10.1371/journal.pone.0233266)
Supplement: S1 Table — Table with stance durations as detected with the algorithms for acceleration, angular velocity and force plate. (DOCX) [file pone.0233266.s003.docx]

**Table S1: Mean and standard deviation of detected stance duration**

| Detected stance duration | | | | |
| --- | --- | --- | --- | --- |
|  |  |  | mean (ms) | SD (ms) |
| acceleration | walk | RF | 779.72 | 50.18 |
|  |  | RH | 777.79 | 53.49 |
|  | trot | RF | 338.85 | 30.94 |
|  |  | RH | 302.63 | 43.63 |
| angular velocity | walk | RF | 786.39 | 53.55 |
|  |  | RH | 784.26 | 48.87 |
|  | trot | RF | 350.77 | 33.00 |
|  |  | RH | 306.32 | 32.89 |
| force plate | walk | RF | 793.06 | 46.46 |
|  |  | RH | 798.68 | 54.11 |
|  | trot | RF | 347.05 | 29.37 |
|  |  | RH | 314.61 | 33.48 |

The mean stance duration and the standard deviation (SD) of this mean in milliseconds (ms) are determined for the acceleration and angular velocity signals, and force plate signal.
