# Supplementary figures and images for: Automatic hoof-on and -off detection in horses using hoof-mounted inertial measurement unit sensors
Source: PLoS One. 2020 Jun 3;15(6):e0233266. doi: 10.1371/journal.pone.0233266 (PMC7269263; doi:10.1371/journal.pone.0233266)

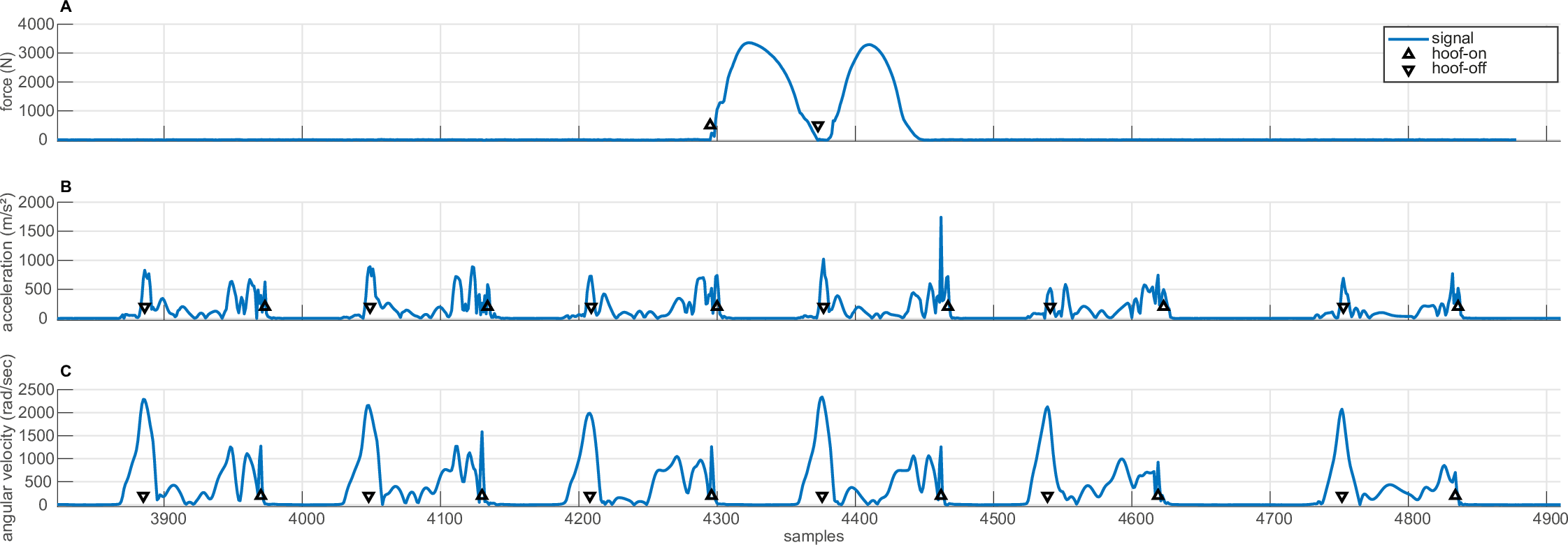

Supplement: S1 Fig — Preprocessed signals of the vertical force (A), the acceleration (B) and angular velocity (C) signals of the IMU for one hoof from one measurement in trot. The hoof-on events are depicted with upward-pointing triangle markers and hoof-off events are depicted with downward-pointing triangle markers. (TIF) [file pone.0233266.s004.tif]
